# Dichlorido(2-{[3-(morpholin-4-ium-4-yl)prop­yl]imino­meth­yl}phenolate)zinc

**DOI:** 10.1107/S1600536811022021

**Published:** 2011-06-18

**Authors:** Nurul Azimah Ikmal Hisham, Hamid Khaledi, Hapipah Mohd Ali

**Affiliations:** a Department of Chemistry, University of Malaya, 50603 Kuala Lumpur, Malaysia

## Abstract

In the zwitterionic zinc title complex, [ZnCl_2_(C_14_H_20_N_2_O_2_)], the Zn^II^ ion is four-coordinated in a distorted tetra­hedral geometry. The Schiff base ligand employs its phenolate O and imine N atoms to coordinate the metal atom in a bidentate mode. Two Cl atoms complete the tetra­hedral coordination environment. In the crystal, a pair of N—H⋯O hydrogen bonds connect the mol­ecules into a centrosymmetric dimer. C—H⋯O, C—H⋯Cl and C—H⋯π inter­actions are also observed.

## Related literature

For related structures of similar zwitterionic ZnCl_2_ complexes, see: Qiu (2006 ▶); Ye & You (2008 ▶); Zhu (2008 ▶).
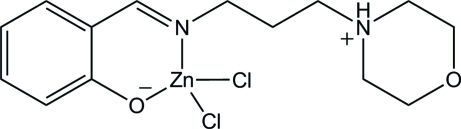


## Experimental

### 

#### Crystal data

[ZnCl_2_(C_14_H_20_N_2_O_2_)]
*M* *_r_* = 384.59
Monoclinic, 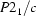

*a* = 8.11276 (10) Å
*b* = 11.21021 (13) Å
*c* = 18.4097 (2) Å
β = 92.0168 (6)°
*V* = 1673.24 (4) Å^3^
*Z* = 4
Mo *K*α radiation
μ = 1.79 mm^−1^
*T* = 100 K
0.37 × 0.32 × 0.25 mm

#### Data collection

Bruker APEXII CCD diffractometer
Absorption correction: multi-scan (*SADABS*; Sheldrick, 1996 ▶) *T*_min_ = 0.557, *T*_max_ = 0.663
14420 measured reflections
3824 independent reflections
3557 reflections with *I* > 2σ(*I*)
*R*_int_ = 0.017

#### Refinement

*R*[*F* ^2^ > 2σ(*F* ^2^)] = 0.020
*wR*(*F* ^2^) = 0.051
*S* = 1.07
3824 reflections
193 parameters
1 restraint
H atoms treated by a mixture of independent and constrained refinement
Δρ_max_ = 0.34 e Å^−3^
Δρ_min_ = −0.33 e Å^−3^

### 

Data collection: *APEX2* (Bruker, 2007 ▶); cell refinement: *SAINT* (Bruker, 2007 ▶); data reduction: *SAINT*; program(s) used to solve structure: *SHELXS97* (Sheldrick, 2008 ▶); program(s) used to refine structure: *SHELXL97* (Sheldrick, 2008 ▶); molecular graphics: *X-SEED* (Barbour, 2001 ▶); software used to prepare material for publication: *SHELXL97* and *publCIF* (Westrip, 2010 ▶).

## Supplementary Material

Crystal structure: contains datablock(s) I, global. DOI: 10.1107/S1600536811022021/is2701sup1.cif

Structure factors: contains datablock(s) I. DOI: 10.1107/S1600536811022021/is2701Isup2.hkl

Additional supplementary materials:  crystallographic information; 3D view; checkCIF report

## Figures and Tables

**Table 1:** Hydrogen-bond geometry (Å, °) *Cg*1 is the centroid of the C1–C6 ring.

*D*—H⋯*A*	*D*—H	H⋯*A*	*D*⋯*A*	*D*—H⋯*A*
N2—H2*N*⋯O1^i^	0.90 (1)	1.81 (1)	2.6954 (14)	170 (2)
C5—H5⋯O2^ii^	0.95	2.39	3.2161 (16)	146
C9—H9*A*⋯Cl1^iii^	0.99	2.83	3.6732 (13)	144
C10—H10*A*⋯Cl1^i^	0.99	2.82	3.6905 (13)	147
C14—H14*A*⋯Cl2^iii^	0.99	2.69	3.5486 (13)	146
C14—H14*B*⋯Cl2^iv^	0.99	2.78	3.6890 (14)	153
C12—H12*B*⋯*Cg*1^v^	0.99	2.57	3.4366 (2)	146

Symmetry codes: (i) 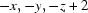; (ii) 
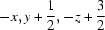
; (iii) 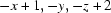; (iv) 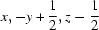; (v) 
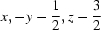
.

**Fig. 1.**
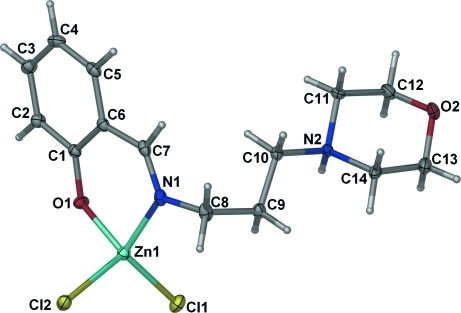
Displacement ellipsoid plot of the title compound at the 50% probability level. Hydrogen atoms are drawn as spheres of arbitrary radius.

## supplementary crystallographic information

### Comment

The title compound was obtained *via* the complexation of ZnCl_2_ with the *in situ* prepared Schiff base. The Schiff base ligand coordinates the metal ion *via* its phenolate oxygen and imine nitrogen atoms. The morpholine ring N atom stays away from the coordination and is protonated, implying the zwitterionic nature of the molecule. The tetrahedral geometry around the zinc(II) ion is completed by two Cl atoms. The coordination bond lengths in the complex are comparable to the corresponding values in similar structures (Qiu, 2006; Ye & You, 2008; Zhu, 2008). In the crystal, N—H···O hydrogen bonding connects pairs of the molecules into centrosymmetric dimers. The dimers are linked through C—H···O, C—H···Cl and C—H···π interactions into a three-dimensional network.

### Experimental

A mixture of salicylaldehyde (0.20 g, 1.64 mmol) and *N*-(3-aminopropyl)morpholine (0.24 g, 1.64 mmol) in ethanol (20 ml) was refluxed for 2 hr followed by addition of a solution of zinc(II) chloride (0.22 g, 1.64 mmol) in a minimum amount of water. The resulting solution was refluxed for 30 min, then the solvent was removed under reduced pressure. The impure product was recrystallized from methanol to give the yellow crystals of the title compound.

### Refinement

The C-bound H atoms were placed at calculated positions at distances C—H = 0.95 and 0.99 Å for aryl and methylene type H-atoms, respectively. The N-bound H atom was placed in a difference Fourier map, and was refined with a distance restraint of N—H 0.91 (2) Å. For all hydrogen atoms *U*_iso_(H) were set to 1.2 times *U*_eq_(carrier atom).

### Crystal data

**Table:**

[ZnCl_2_(C_14_H_20_N_2_O_2_)]	*F*(000) = 792
*M**_r_* = 384.59	*D*_x_ = 1.527 Mg m^−^^3^
Monoclinic, *P*2_1_/*c*	Mo *K*α radiation, λ = 0.71073 Å
Hall symbol: -P 2ybc	Cell parameters from 9373 reflections
*a* = 8.11276 (10) Å	θ = 2.2–30.4°
*b* = 11.21021 (13) Å	µ = 1.79 mm^−^^1^
*c* = 18.4097 (2) Å	*T* = 100 K
β = 92.0168 (6)°	Block, yellow
*V* = 1673.24 (4) Å^3^	0.37 × 0.32 × 0.25 mm
*Z* = 4	

### Data collection

**Table:**

Bruker APEXII CCD diffractometer	3824 independent reflections
Radiation source: fine-focus sealed tube	3557 reflections with *I* > 2σ(*I*)
graphite	*R*_int_ = 0.017
φ and ω scans	θ_max_ = 27.5°, θ_min_ = 2.1°
Absorption correction: multi-scan (*SADABS*; Sheldrick, 1996)	*h* = −10→10
*T*_min_ = 0.557, *T*_max_ = 0.663	*k* = −14→14
14420 measured reflections	*l* = −23→23

### Refinement

**Table:**

Refinement on *F*^2^	Primary atom site location: structure-invariant direct methods
Least-squares matrix: full	Secondary atom site location: difference Fourier map
*R*[*F*^2^ > 2σ(*F*^2^)] = 0.020	Hydrogen site location: inferred from neighbouring sites
*wR*(*F*^2^) = 0.051	H atoms treated by a mixture of independent and constrained refinement
*S* = 1.07	*w* = 1/[σ^2^(*F*_o_^2^) + (0.0237*P*)^2^ + 0.6991*P*] where *P* = (*F*_o_^2^ + 2*F*_c_^2^)/3
3824 reflections	(Δ/σ)_max_ = 0.003
193 parameters	Δρ_max_ = 0.34 e Å^−^^3^
1 restraint	Δρ_min_ = −0.33 e Å^−^^3^

### Special details

**Table:**

Geometry. All e.s.d.'s (except the e.s.d. in the dihedral angle between two l.s. planes) are estimated using the full covariance matrix. The cell e.s.d.'s are taken into account individually in the estimation of e.s.d.'s in distances, angles and torsion angles; correlations between e.s.d.'s in cell parameters are only used when they are defined by crystal symmetry. An approximate (isotropic) treatment of cell e.s.d.'s is used for estimating e.s.d.'s involving l.s. planes.
Refinement. Refinement of *F*^2^ against ALL reflections. The weighted *R*-factor *wR* and goodness of fit *S* are based on *F*^2^, conventional *R*-factors *R* are based on *F*, with *F* set to zero for negative *F*^2^. The threshold expression of *F*^2^ > σ(*F*^2^) is used only for calculating *R*-factors(gt) *etc*. and is not relevant to the choice of reflections for refinement. *R*-factors based on *F*^2^ are statistically about twice as large as those based on *F*, and *R*- factors based on ALL data will be even larger.

### Fractional atomic coordinates and isotropic or equivalent isotropic displacement parameters (Å^2^)

**Table:**

	*x*	*y*	*z*	*U*_iso_*/*U*_eq_	
Zn1	0.187917 (17)	0.093296 (13)	1.107214 (8)	0.01475 (5)	
Cl1	0.22466 (4)	−0.09919 (3)	1.084342 (19)	0.02075 (8)	
Cl2	0.33968 (4)	0.15593 (3)	1.205005 (18)	0.01907 (7)	
O1	−0.04186 (11)	0.13291 (8)	1.12739 (5)	0.01608 (18)	
O2	0.13093 (14)	−0.02283 (10)	0.65039 (5)	0.0276 (2)	
N1	0.20412 (13)	0.20247 (10)	1.02150 (6)	0.0159 (2)	
N2	0.16476 (13)	0.05293 (10)	0.79901 (6)	0.0135 (2)	
H2N	0.1344 (19)	−0.0140 (12)	0.8218 (8)	0.016*	
C1	−0.12380 (15)	0.22475 (11)	1.09867 (7)	0.0137 (2)	
C2	−0.28113 (16)	0.25169 (12)	1.12420 (7)	0.0163 (2)	
H2	−0.3253	0.2032	1.1611	0.020*	
C3	−0.37327 (16)	0.34666 (12)	1.09714 (7)	0.0186 (3)	
H3	−0.4787	0.3627	1.1158	0.022*	
C4	−0.31222 (17)	0.41883 (12)	1.04277 (8)	0.0196 (3)	
H4	−0.3743	0.4849	1.0247	0.024*	
C5	−0.16034 (17)	0.39287 (12)	1.01564 (7)	0.0173 (3)	
H5	−0.1203	0.4405	0.9774	0.021*	
C6	−0.06264 (15)	0.29814 (11)	1.04276 (7)	0.0142 (2)	
C7	0.09231 (16)	0.28065 (11)	1.00702 (7)	0.0159 (2)	
H7	0.1134	0.3336	0.9682	0.019*	
C8	0.34713 (16)	0.19638 (13)	0.97446 (7)	0.0194 (3)	
H8A	0.4473	0.1769	1.0044	0.023*	
H8B	0.3641	0.2753	0.9518	0.023*	
C9	0.32253 (16)	0.10212 (12)	0.91483 (7)	0.0170 (3)	
H9A	0.4296	0.0847	0.8929	0.020*	
H9B	0.2812	0.0274	0.9363	0.020*	
C10	0.20034 (15)	0.14566 (11)	0.85628 (7)	0.0152 (2)	
H10A	0.0960	0.1682	0.8790	0.018*	
H10B	0.2452	0.2178	0.8331	0.018*	
C11	0.02330 (16)	0.09176 (11)	0.74944 (7)	0.0171 (3)	
H11A	0.0509	0.1679	0.7255	0.020*	
H11B	−0.0763	0.1045	0.7780	0.020*	
C12	−0.01012 (18)	−0.00338 (13)	0.69264 (7)	0.0224 (3)	
H12A	−0.0405	−0.0787	0.7168	0.027*	
H12B	−0.1042	0.0214	0.6604	0.027*	
C13	0.26442 (19)	−0.06439 (14)	0.69587 (8)	0.0253 (3)	
H13A	0.3610	−0.0801	0.6659	0.030*	
H13B	0.2328	−0.1403	0.7190	0.030*	
C14	0.31118 (16)	0.02596 (12)	0.75431 (7)	0.0180 (3)	
H14A	0.4018	−0.0062	0.7860	0.022*	
H14B	0.3505	0.1002	0.7315	0.022*	

### Atomic displacement parameters (Å^2^)

**Table:**

	*U*^11^	*U*^22^	*U*^33^	*U*^12^	*U*^13^	*U*^23^
Zn1	0.01268 (8)	0.01573 (8)	0.01592 (9)	0.00041 (5)	0.00142 (6)	−0.00096 (5)
Cl1	0.01746 (15)	0.01744 (15)	0.02752 (17)	0.00001 (11)	0.00307 (13)	−0.00615 (12)
Cl2	0.01577 (14)	0.02111 (16)	0.02017 (16)	0.00145 (11)	−0.00147 (11)	−0.00506 (12)
O1	0.0137 (4)	0.0163 (4)	0.0184 (5)	0.0015 (3)	0.0024 (3)	0.0039 (4)
O2	0.0390 (6)	0.0322 (6)	0.0117 (5)	0.0108 (5)	0.0007 (4)	−0.0017 (4)
N1	0.0148 (5)	0.0191 (5)	0.0140 (5)	−0.0042 (4)	0.0023 (4)	−0.0036 (4)
N2	0.0153 (5)	0.0139 (5)	0.0114 (5)	0.0016 (4)	0.0022 (4)	0.0009 (4)
C1	0.0147 (6)	0.0137 (6)	0.0127 (6)	−0.0014 (4)	−0.0014 (4)	−0.0014 (4)
C2	0.0153 (6)	0.0170 (6)	0.0165 (6)	−0.0005 (5)	0.0010 (5)	0.0012 (5)
C3	0.0137 (6)	0.0202 (6)	0.0219 (7)	0.0011 (5)	−0.0010 (5)	−0.0014 (5)
C4	0.0192 (6)	0.0171 (6)	0.0220 (7)	0.0014 (5)	−0.0066 (5)	0.0019 (5)
C5	0.0219 (6)	0.0165 (6)	0.0134 (6)	−0.0039 (5)	−0.0035 (5)	0.0008 (5)
C6	0.0164 (6)	0.0139 (6)	0.0121 (6)	−0.0019 (4)	−0.0007 (5)	−0.0016 (4)
C7	0.0205 (6)	0.0157 (6)	0.0117 (6)	−0.0056 (5)	0.0011 (5)	−0.0013 (5)
C8	0.0141 (6)	0.0269 (7)	0.0173 (6)	−0.0057 (5)	0.0035 (5)	−0.0040 (5)
C9	0.0135 (6)	0.0222 (7)	0.0154 (6)	0.0004 (5)	0.0008 (5)	−0.0025 (5)
C10	0.0165 (6)	0.0150 (6)	0.0142 (6)	0.0001 (5)	0.0018 (5)	−0.0019 (5)
C11	0.0183 (6)	0.0189 (6)	0.0138 (6)	0.0042 (5)	−0.0017 (5)	0.0014 (5)
C12	0.0284 (7)	0.0240 (7)	0.0143 (6)	0.0020 (6)	−0.0042 (5)	0.0000 (5)
C13	0.0333 (8)	0.0257 (7)	0.0169 (7)	0.0105 (6)	0.0037 (6)	−0.0020 (5)
C14	0.0199 (6)	0.0194 (6)	0.0153 (6)	0.0055 (5)	0.0065 (5)	0.0020 (5)

### Geometric parameters (Å, °)

**Table:**

Zn1—O1	1.9644 (9)	C5—H5	0.9500
Zn1—N1	2.0049 (11)	C6—C7	1.4526 (18)
Zn1—Cl1	2.2206 (3)	C7—H7	0.9500
Zn1—Cl2	2.2570 (3)	C8—C9	1.5318 (18)
O1—C1	1.3254 (15)	C8—H8A	0.9900
O2—C12	1.4230 (17)	C8—H8B	0.9900
O2—C13	1.4238 (18)	C9—C10	1.5190 (18)
N1—C7	1.2825 (17)	C9—H9A	0.9900
N1—C8	1.4736 (16)	C9—H9B	0.9900
N2—C14	1.4995 (16)	C10—H10A	0.9900
N2—C10	1.5012 (16)	C10—H10B	0.9900
N2—C11	1.5052 (16)	C11—C12	1.5114 (18)
N2—H2N	0.898 (13)	C11—H11A	0.9900
C1—C2	1.4084 (17)	C11—H11B	0.9900
C1—C6	1.4206 (17)	C12—H12A	0.9900
C2—C3	1.3836 (18)	C12—H12B	0.9900
C2—H2	0.9500	C13—C14	1.516 (2)
C3—C4	1.392 (2)	C13—H13A	0.9900
C3—H3	0.9500	C13—H13B	0.9900
C4—C5	1.377 (2)	C14—H14A	0.9900
C4—H4	0.9500	C14—H14B	0.9900
C5—C6	1.4062 (18)		
			
O1—Zn1—N1	95.72 (4)	C9—C8—H8A	109.3
O1—Zn1—Cl1	112.96 (3)	N1—C8—H8B	109.3
N1—Zn1—Cl1	115.53 (3)	C9—C8—H8B	109.3
O1—Zn1—Cl2	105.87 (3)	H8A—C8—H8B	108.0
N1—Zn1—Cl2	112.88 (3)	C10—C9—C8	110.63 (11)
Cl1—Zn1—Cl2	112.363 (13)	C10—C9—H9A	109.5
C1—O1—Zn1	124.48 (8)	C8—C9—H9A	109.5
C12—O2—C13	109.77 (10)	C10—C9—H9B	109.5
C7—N1—C8	118.38 (11)	C8—C9—H9B	109.5
C7—N1—Zn1	121.01 (9)	H9A—C9—H9B	108.1
C8—N1—Zn1	120.60 (9)	N2—C10—C9	112.36 (10)
C14—N2—C10	112.86 (10)	N2—C10—H10A	109.1
C14—N2—C11	109.11 (10)	C9—C10—H10A	109.1
C10—N2—C11	110.44 (10)	N2—C10—H10B	109.1
C14—N2—H2N	108.8 (10)	C9—C10—H10B	109.1
C10—N2—H2N	107.5 (10)	H10A—C10—H10B	107.9
C11—N2—H2N	108.0 (10)	N2—C11—C12	109.25 (10)
O1—C1—C2	118.76 (11)	N2—C11—H11A	109.8
O1—C1—C6	123.77 (11)	C12—C11—H11A	109.8
C2—C1—C6	117.47 (11)	N2—C11—H11B	109.8
C3—C2—C1	121.96 (12)	C12—C11—H11B	109.8
C3—C2—H2	119.0	H11A—C11—H11B	108.3
C1—C2—H2	119.0	O2—C12—C11	110.99 (12)
C2—C3—C4	120.32 (13)	O2—C12—H12A	109.4
C2—C3—H3	119.8	C11—C12—H12A	109.4
C4—C3—H3	119.8	O2—C12—H12B	109.4
C5—C4—C3	118.93 (12)	C11—C12—H12B	109.4
C5—C4—H4	120.5	H12A—C12—H12B	108.0
C3—C4—H4	120.5	O2—C13—C14	111.40 (11)
C4—C5—C6	122.09 (12)	O2—C13—H13A	109.3
C4—C5—H5	119.0	C14—C13—H13A	109.3
C6—C5—H5	119.0	O2—C13—H13B	109.3
C5—C6—C1	119.19 (12)	C14—C13—H13B	109.3
C5—C6—C7	115.28 (12)	H13A—C13—H13B	108.0
C1—C6—C7	125.44 (12)	N2—C14—C13	109.93 (11)
N1—C7—C6	127.96 (12)	N2—C14—H14A	109.7
N1—C7—H7	116.0	C13—C14—H14A	109.7
C6—C7—H7	116.0	N2—C14—H14B	109.7
N1—C8—C9	111.57 (10)	C13—C14—H14B	109.7
N1—C8—H8A	109.3	H14A—C14—H14B	108.2

### Hydrogen-bond geometry (Å, °)

**Table:**

*Cg*1 is the centroid of the C1–C6 ring.

**Table:**

*D*—H···*A*	*D*—H	H···*A*	*D*···*A*	*D*—H···*A*
N2—H2N···O1^i^	0.90 (1)	1.81 (1)	2.6954 (14)	170.(2)
C5—H5···O2^ii^	0.95	2.39	3.2161 (16)	146
C9—H9A···Cl1^iii^	0.99	2.83	3.6732 (13)	144
C10—H10A···Cl1^i^	0.99	2.82	3.6905 (13)	147
C14—H14A···Cl2^iii^	0.99	2.69	3.5486 (13)	146
C14—H14B···Cl2^iv^	0.99	2.78	3.6890 (14)	153
C12—H12B···Cg1^v^	0.99	2.57	3.43663 (15)	146

Symmetry codes: (i) −*x*, −*y*, −*z*+2; (ii) −*x*, *y*+1/2, −*z*+3/2; (iii) −*x*+1, −*y*, −*z*+2; (iv) *x*, −*y*+1/2, *z*−1/2; (v) *x*, −*y*−1/2, *z*−3/2.
